# Spatial Overlap between Environmental Policy Instruments and Areas of High Conservation Value in Forest

**DOI:** 10.1371/journal.pone.0115001

**Published:** 2014-12-11

**Authors:** Anne Sverdrup-Thygeson, Gunnhild Søgaard, Graciela M. Rusch, David N. Barton

**Affiliations:** 1 Norwegian University of Life Sciences, Department of Ecology and Natural Resource Management, Aas, Norway; 2 Norwegian Institute for Nature Research (NINA), Oslo, Norway; 3 Norwegian Forest and Landscape Institute (NFLI), Ås, Norway; 4 Norwegian Institute for Nature Research, Trondheim, Norway; University of Waikato (National Institute of Water and Atmospheric Research), New Zealand

## Abstract

In order to safeguard biodiversity in forest we need to know how forest policy instruments work. Here we use a nationwide network of 9400 plots in productive forest to analyze to what extent large-scale policy instruments, individually and together, target forest of high conservation value in Norway. We studied both instruments working through direct regulation; Strict Protection and Landscape Protection, and instruments working through management planning and voluntary schemes of forest certification; Wilderness Area and Mountain Forest. As forest of high conservation value (HCV-forest) we considered the extent of 12 Biodiversity Habitats and the extent of Old-Age Forest. We found that 22% of productive forest area contained Biodiversity Habitats. More than 70% of this area was not covered by any large-scale instruments. Mountain Forest covered 23%, while Strict Protection and Wilderness both covered 5% of the Biodiversity Habitat area. A total of 9% of productive forest area contained Old-Age Forest, and the relative coverage of the four instruments was similar as for Biodiversity Habitats. For all instruments, except Landscape Protection, the targeted areas contained significantly higher proportions of HCV-forest than areas not targeted by these instruments. Areas targeted by Strict Protection had higher proportions of HCV-forest than areas targeted by other instruments, except for areas targeted by Wilderness Area which showed similar proportions of Biodiversity Habitats. There was a substantial amount of spatial overlap between the policy tools, but no incremental conservation effect of overlapping instruments in terms of contributing to higher percentages of targeted HCV-forest. Our results reveal that although the current policy mix has an above average representation of forest of high conservation value, the targeting efficiency in terms of area overlap is limited. There is a need to improve forest conservation and a potential to cover this need by better targeting high conservation value areas.

## Introduction

Forests are diverse systems, representing some of the richest biological areas on Earth. While timber production used to be regarded as the dominant function of forests, other ecosystem services such as provision of opportunities for recreation, maintenance of biological diversity, and the role of forest in climate regulation are increasingly recognized as integral components of sustainable forest management.

In most countries, the sustainable use and conservation of forest builds on strategies involving a wide range of policy instruments, ranging from direct regulation and spatial planning, via economic instruments like biodiversity offsets, environmental taxes or tax reliefs, ecological fiscal transfers and payments for environmental services (PES), to voluntary approaches like forest certification. In practical politics, several instruments from these categories can often be found in combination, creating a policy mix [1]. This could result in adverse effects - if one instrument counteracts the effect of another - or in redundancy and inefficient use of resources - if several policy tools address the same objective. On the other hand, a mix of spatially complementary and synergistic policy instruments might be effective in the complex and multi-targeted task of protecting all ecosystem services across heterogeneous forest landscapes [1], [2]. This is especially true for the challenges of biodiversity protection.

In Norway, the legal and regulatory frameworks to protect biodiversity have gradually been strengthened in the past 20 years. Protected Areas in the form of nature reserves, national parks and landscape protection are regulated through the Nature Diversity Act from 2009, while both the Nature Diversity Act and the Forestry Act include measures that promote sustainable use of the remaining areas. The Forestry Act regulates special treatment of forest on mountain slopes and an amendment from 2006 regulates the delineation of smaller patches consisting of habitats that are important for forest biodiversity conservation (Woodland Key Habitats; average size in Norway is 1 ha [3]). The implementation of the Woodland Key Habitats as well as certain restrictions in high-altitude forest is described in the two operational forest certification guidelines in Norway (PEFC Norway Forest Management Standard and versions of the generic Forest Stewardship Council's Standard). Almost all Norwegian forest is presently certified with the PEFC standard. The certification scheme also regulates retention of trees and buffer zones along waterways, as well as avoidance of clear-cutting in swamp forest.

Despite the strengthening of the conservation instruments, the Norwegian Office of the Auditor General (OAG) recently concluded that based on available information it is difficult to provide an overall assessment of the status of forest biodiversity conservation and recreational values, and also of how forestry safeguards environmental requirements [4]. The OAG review also revealed potential conflicts between forestry and conservation objectives. This motivates a further inquiry, investigating both the efficiency of the policy instruments in targeting areas of high conservation value, and the extent to which the different regulatory and voluntary environmental policy instruments at work in Norwegian forests are complementary or redundant.

To evaluate quantitatively the impact of conservation measures is a challenge. Previous studies of impact evaluation have often used forest cover as a proxy for biodiversity conservation gains, and not indicators of forest quality [5], [6]. Even though attempts have been made to create and use biodiversity indicators at national level [2],[7], the spatial resolution has been too coarse to assess the spatial occurrence and spatial overlap among different types of regulations and areas of high biodiversity value compared to what is needed for forestry and conservation planning. We have some information on the relative conservation outcomes of different conservation measures in boreal forest, but most focus on small-scale conservation measures related to harvested areas [8]–[10]. For the large-scale policy instruments in Norwegian forest we don't even know their relative success in targeting known areas of high biodiversity value, let alone their effectiveness in safeguarding these in a long term perspective.

In this study we take a step further by using data from the National Forest Inventory (NFI), consisting of spatially explicit and representative data on forest conditions and certain measures of biodiversity values covering all productive forest in Norway. We use the occurrences of *Biodiversity Habitats* and *Old-Age Forest* as indicators of forest of high conservation value (HCV forest [11], [12]). We combine these data with the spatial extent of the main policy instruments addressing large-scale environmental conservation in Norwegian forest. Some of these instruments work through direct regulation (Strict Protection and Landscape Protection Area), and some work through management planning processes and voluntary certification schemes (Wilderness Area and Mountain Forest). We ask the following research questions:

- To what extent do areas targeted by different forest conservation instruments coincide with areas of high conservation value in Norwegian productive forest?
- Do different kinds of policy instruments complement each other in targeting different biodiversity features?

While forestry is banned in Strict Protection areas, forestry is permitted with different limitations under the three other instruments. Our main hypothesis was that the proportion of Biodiversity Habitats and Old-Age Forest would be higher in areas with higher restrictions. We further tested overlap, both between policy instruments and our two indicators on high conservation value.

## Materials and Methods

### The NFI data set

The material consisted of data collected on a systematic network of permanent sampling plots from the Norwegian National Forest Inventory (NFI). The data set covers the whole country except the northernmost county, Finnmark (dominated by tundra), with two grid systems, a 3×3 km grid below, and a 3×9 km grid above the timber line. In this study we focused on forest areas with sufficient production potential for forestry (1 m^3^/ha/year, [13]), called productive forest, since these are the areas where trade-offs between timber production and conservation are most likely to occur. This included 9431 NFI plots, representing the state of all Norwegian productive forest.

The NFI permanent plots are circular with radius  = 8.92 m, for which more than 60 tree and stand variables were measured. In addition, an extended plot of 25 m radius is used for an inventory of 12 habitats considered important for red-listed species and thus for forest biodiversity conservation, here shortened to Biodiversity Habitats (described in more detail below). If one or more Biodiversity Habitats occur in the extended plot area, the surroundings are surveyed to assess whether the total area of each habitat exceeds the minimum size of 0.2 ha required by the field protocol. If so, the proportion of the extended plot covered by the habitat is recorded. As the sampling is large and representative, these data can be used to calculate the proportion of Biodiversity Habitats in all productive forest.

One-fifth of the permanent plots in the NFI are measured every year, resulting in complete re-measurement in a 5-year cycle. For our study, we used data from the period 2005–2009. A previous report, in Norwegian [14], used the same dataset to analyze the aggregated effect of all environmental and recreational considerations on the available area of productive forest, and on the proportion of growing stock that can be harvested. Here, we focus on the policy instruments addressing *large-scale* environmental conservation in Norwegian forest. We do not include set aside areas in the form of retention patches and woodland key habitats, as these are small (average size of woodland key habitats is 1 ha in Norway [3]) and have a different role in biodiversity conservation than large protected areas [15], [16].

For estimates on representation, means and 95% confidence intervals were generated by stratified bootstrap, with 2000 replicates accounting for the sampling error [17]. For two-way comparison we used non-parametric analysis with Wilcoxon Rank Sum. All analyses where carried out in R version 3.0.1 [18].

Two different indicators of forest of high conservation value were used; the occurrence of *Biodiversity Habitats* and of *Old-Age Forest*:

### Biodiversity Habitats

Twelve types of forest habitats of particular importance for red-listed forest species [19] are recorded as part of the NFI. These habitats are: snags, logs, trees with nutrient-rich bark, trees with pendant lichens, late successional forests with deciduous trees, old trees, hollow deciduous trees, recently burned forest, luxuriant ground vegetation, rock walls, clay ravines and stream gorges (**Table 1**). The habitats were defined in the same way as in the Complementary Hotspot Inventory conducted in regional forestry planning in Norway [19], [20]. The habitats rock walls, clay ravines and stream gorges are only registered on a presence/absence level and hollow deciduous trees are only recorded as points. Therefore these habitats cannot be included in area estimates, but are included when the number of co-occurring habitats is calculated (see below). With the exception of rock walls, these habitats have a very low frequency.

**Table 1:** Description of the Biodiversity Habitats included in the study.

Biodiversity Habitats	Survey method and criteria (all area-based habitats >0.2 ha)
Snags	Area with minimum 40 stems/ha (for 10–30 cm DBH), 20 stems/ha (for >30 cm DBH)
Logs	Area with minimum 40 stems/ha (for 10–30 cm DBH), 20 stems/ha (for >30 cm DBH)
Trees with nutrient-rich bark	Area with minimum 20–40 trees/ha (depending on district of Norway) with presence of *Lobaria* lichens
Trees with pendant lichens	Area with minimum 100 lichen-rich trees/ha
Late successional broadleaf forest	Area with minimum 40 boreal broadleaf trees >20 cm DBH/ha
Old trees	Area with minimum 30 old trees/ha (diameter criteria for different broadleaf trees; visual characteristics for coniferous trees, corresponding to age >150–200 yrs)
Hollow deciduous trees	Occurrence (point coordinates) of deciduous trees with rot cavities
Burned forest	Area with recently burned (<10 years ago) forest with standing dead wood
Rich ground vegetation	Area with selected rich vegetation types, depending on district in Norway
Rock walls	Occurrence of rock walls >3 m height and >60% incline
Clay ravines	Occurrence of clay ravines >25 m in length
Stream gorges	Occurrence of stream gorges more than 5 m deep and more than 25 m in length

Different Biodiversity Habitats may be present in the same plot, with no, partial or full spatial overlap. Information on the proportion of the plot area that each habitat covers was available, but not on the extent to which they overlap. Previous studies have shown that the degree of spatial overlap between co-occurring habitats varies between areas [21]. Here we calculated the area covered by Biodiversity Habitats as the mean between the maximum and the minimum spatial extent, where maximum Biodiversity Habitat extent was the sum of the areas of all Biodiversity Habitats in the same plot, and minimum Biodiversity Habitat was the area of the largest Biodiversity Habitat.

Areas with several spatially overlapping or adjoining Biodiversity Habitats represent high concentrations of complementary conservation features and can be considered as especially important. We conducted a separate analysis of this subset only, called *Multi-Biodiversity Habitats*. We also did this for the subset of Biodiversity Habitats with spatial overlap with Old-Age Forest (see below), as *Old-Age Biodiversity Habitats*.

### Old-Age forest

Old-Age Forest is defined as forest significantly above harvesting age, adjusting for tree species and site index (i.e. forest productivity) [22] (**Table 2**). This is based on the data recorded on all 8.92 m sample plots. Old-Age Forest is defined only by high age and does not include any criteria on the degree of human intervention (logging, planting etc.). Currently, Old-Age Forest per se is not protected under any special regulation.

**Table 2:** Minimum age for definition as Old-Age Forest, based on site index and tree species.

Site productivity index	Broad leaf forest	Spruce-dominated forest (*Picea abies*)	Pine-dominated forest (*Pinus sylvestris*)
Low	120 years	160 years	180 years
Medium-high	100 years	140 years	160 years
High-very high	80 years	120 years	140 years

This corresponds to the age thresholds applied in the baseline work of the Norwegian Nature Index [22], [38].

### Policy instruments regulating forest use

The Norwegian Nature Diversity Act defines four classes of policy instruments in the form of protected areas relevant for the study: Nature reserve, national park, biotope protection and protected landscape area (**Table 3**).

**Table 3:** Description of the policy mix included in the study.

Policy tool	Conservation level/multiple use, objectives	Regulated by
**Strict Protection**	**National park** (Absolute conservation, no forest management allowed. Protect larger areas and ecosystems) and **nature reserve** (Absolute conservation, no forest management allowed. Protect areas of special biodiversity value).	Nature Diversity Act
**Landscape Protection**	**Protected landscape area** (Forestry permitted under certain circumstances. Protect landscapes of ecological or cultural value) and **biotope protection** (Forestry permitted under certain circumstances. Protect specific species and their biotopes).	Nature Diversity Act
**Wilderness Area**	Forestry permitted but forest road building not subsidized.	Forestry Act + Forest certification
**Mountain Forest**	Multiple use forest. Forestry is secondary to other forest services (climate control, nature hazard control + biodiversity, recreation).	Forestry Act + Forest certification

Nature reserves have the strictest level of protection, while protected landscape areas has the least strict. Nature reserves are established on areas that have natural values that are threatened, rare or vulnerable, that can represent a particular type of nature, or that have particular importance for biodiversity protection or for research. National parks are aimed at protecting larger areas of ecosystems or landscapes that have comparatively limited anthropogenic intervention and that are representative of a particular ecosystem type and/or that have particular natural features. As the extent of forest in national parks is limited, and the restriction level concerning forestry is similar to nature reserves, these two classes are merged and called **Strict Protection**.

Landscape protection aims to protect landscapes of ecological or cultural value. Land-use forms at the time of establishment, such as logging, can continue, but often with certain restrictions. Biotope protection areas are intended to protect specific species and their biotopes, and are usually small areas with different restriction levels depending on the need of the species. As the dataset only contained two plots in biotope protection areas, and the restriction level is comparable with regard to forestry activities, this class is included under landscape protection and denoted **Landscape Protection**.

We also considered two use regimes that partially protect forest: Mountain Forest and Wilderness Area. **Mountain Forest** (**Table 3**) is defined according to The Forestry Act [23]. Forests on slopes (i.e., near the non-forested alpine zone) are vulnerable. They may also serve as protection from natural hazards, and it may protect the forest below from local adverse climate effects. Determining both the boundaries and the exact management restrictions in mountain forest is the responsibility of local administrative authorities. The forest certification guidelines also target mountain forest, stating that at least 50% of the mountain forest should have a mature forest character, to ensure their biodiversity and recreational values [12].

We also included **Wilderness Area** as a conservation instrument, defined as areas located more than 5 km from the nearest significant technical intervention in the landscape and a surrounding buffer zone 4.5 km wide (**Table 3**) [24], [25]. Logging is permitted within Wilderness Areas and their buffer zones. Still, according to current directions under the Forestry Act, subsidies for permanent road building will not to be granted where they would reduce the extent of the wilderness area [26]. Therefore, logging is seldom profitable in these areas [27].

Geo-referenced NFI data were combined with digitized maps and suplementary information of protected areas, Wilderness Area and Mountain Forest to determine the spatial overlap.

### Ethics statement and data accessibility

No specific permission was required for field work in any of the locations, and the field study did not involve endangered or protected species. All data were collected as part of the ordinary NFI forest monitoring, by NFI staff. The field plots are located in a 3×3 km grid over the whole country (>9400 plots visited). Exact location of field plots (GPS-coordinates) are confidential to ensure objective treatment of the forest at each location and thus the integrity of the data gathered for monitoring purposes. All data used in the study are accessible from the Norwegian Forest and Landscape Institute. The data are held in a public repository, partly open online access (http://www.skogoglandskap.no/temaer/statistikk_fra_landsskogstakseringen) and partly upon request.

## Results

### Forest with high conservation value

**Figure 1:**
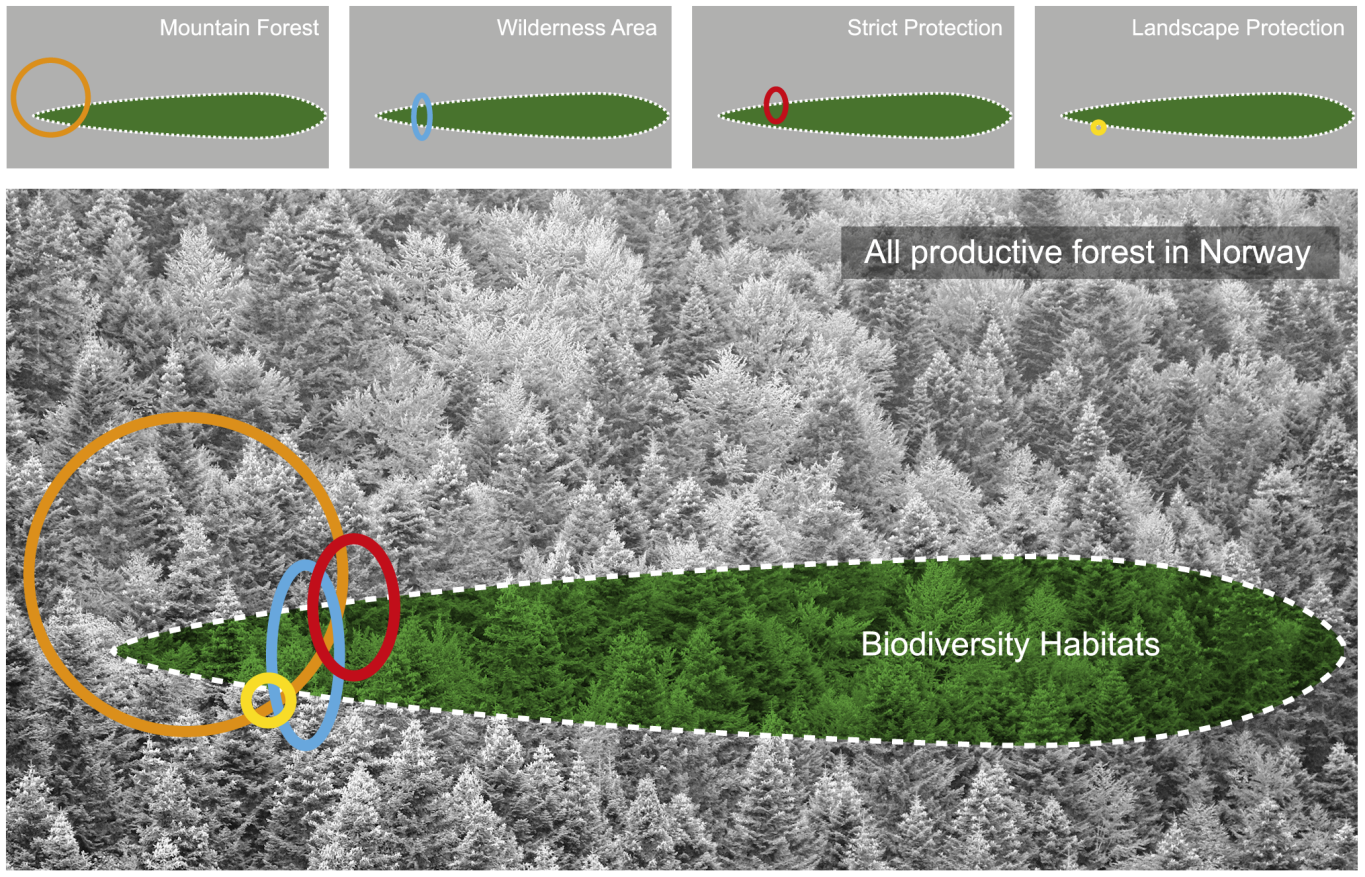
Illustration of the scale of overlap and targeting effectiveness of main large-scale policy instruments in Norwegian forest, for habitats of importance for red-listed species (Biodiversity Habitats). The large grey square of forest illustrates all productive forest in Norway, while the green leaf shape symbolizes the 22% of productive forest with Biodiversity Habitats. The four large-scale policy instruments are symbolized as follows: Orange circle for Mountain Forest (17% of Prod. forest, 23% of Biodiversity Habitat), Blue ellipsoid for Wilderness Area (3% of Prod. forest, 5% of Biodiversity Habitats), Red ellipsoid for Strict protection (2,7% of Prod. forest, 4,9% of Biodiversity Habitats) and Yellow circle for Landscape Protection (1,4% of Prod. forest, 1,6% of Biodiversity Habitats). Old-Age Forest is not included in the figure. Scale is approximate.

The Biodiversity Habitats covered 22% (1 814 493 ha) of Norwegian productive forest area (**Fig. 1**), of which half had two or more habitats present (**Table 4**). The dominating Biodiversity Habitat feature was Logs, which was present in 13.4% of the Norwegian productive forest area. Habitats with snags, trees with pendant lichens and rich ground vegetation each made up around 2.7% of the productive forest area, while the remaining habitats covered less than 2% of productive forest area each (**Table 4**). Hollow deciduous trees, estimated as points, extrapolate to 150 000 hollow trees (95% CI: 89 000-224 000) in all productive forest area in Norway.

**Table 4:** Proportion of productive forest covered by each of the Biodiversity Habitats and by Old-Age Forest.

Forest of high conservation value	Prop. of Norw. productive forest	95% Confidence Interval
Biodiversity Habitats	22,3%	[22,1–22,4]
Individual habitats*:		
Snags	2,7%	[2,7–2,7]
Logs	13,4%	[13,3–13,5]
Trees with nutrient-rich bark	0,2%	[0,2–0,2]
Trees with pendant lichens	2,8%	[2,8–2,8]
Late successional broadleaf forest	1,5%	[1,5–1,5]
Old trees	1,7%	[1,7–1,7]
Burned forest	0,1%	[0,1–0,1]
Rich ground vegetation	2,7%	[2,7–2,7]
Multi-Biodiversity Habitats	10%	[10,0–10,1]
Old-Age Biodiversity Habitats	4,6%	[4,6–4,6]
Old-Age Forest	9,4%	[9,4–9,5]

*Only possible to calculate for the habitats with areal extent, cf. Methods.

Old-Age Forest covered 9.4% (767 000 ha) of Norwegian productive forest (**Table 4**). There was substantial spatial overlap between the Old-Age Forest and the Biodiversity Habitats: 40% of the area in Old-Age Forest overlapped with that of one or more important habitats, as opposed to 17% spatial overlap with Biodiversity Habitats in the remaining forest. Of the total area of Biodiversity Habitats, 19% was in Old-Age Forest, giving a proportion of Old-Age Biodiversity Habitats of 4.6% of productive forest (**Table 4**).

### Main instruments

For productive forest in general, 2.7% (217 000 ha) of the area was situated within an area targeted by Strict Protection, and an additional 1.4% of productive forest was within Landscape Protection areas. In terms of extent, Mountain Forest was the instrument covering the absolutely largest area of productive forest, as much as 17%, while Wilderness Area covered 3% of productive forest (**Fig. 1**, **Table 5**).

**Table 5:** Proportion of forest (productive forest, Biodiversity Habitats and Old-Age Forest) overlapping with different policy instruments.

	Strict Protection	Landscape Protection	Wilderness	Mountain Forest	None of these instruments
Prod. forest	2,7% [2,3–3,1]	1,4% [1,1–1,7]	3,1% [2,6–3,5]	17,1% [16,2–18,0]	80,0% [79,1–81,0]
Biodiversity Habitat	4,9% [3,8–6,0]	1,6% [1,0–2,4]	4,9% [3,8–6,1]	22,6% [20,5–24,9]	72,5% [70,3–74,8]
Old-Age Forest	6,8% [5,1–8,5]	0,5% [0,1–1,0]	4,8% [3,4–6,3]	22,6% [19,7–25,5]	71,7% [68,7–74,7]

95% Confidence Interval given in brackets.

### How do the instruments target areas of high conservation value?

The instruments considered coincided with only a limited proportion of Old-Age Forest and Biodiversity Habitat area, with the exception of Mountain Forest, which covered around 23% of both (**Table 5**). A similar proportion (5%) of Biodiversity Habitats was found within Wilderness Areas as within Strict Protection areas. This was also the case for Old-Age Forest (**Table 5**). More than 70% of the area of known forest with high conservation value was not covered by any of the studied instruments.

**Figure 2:**
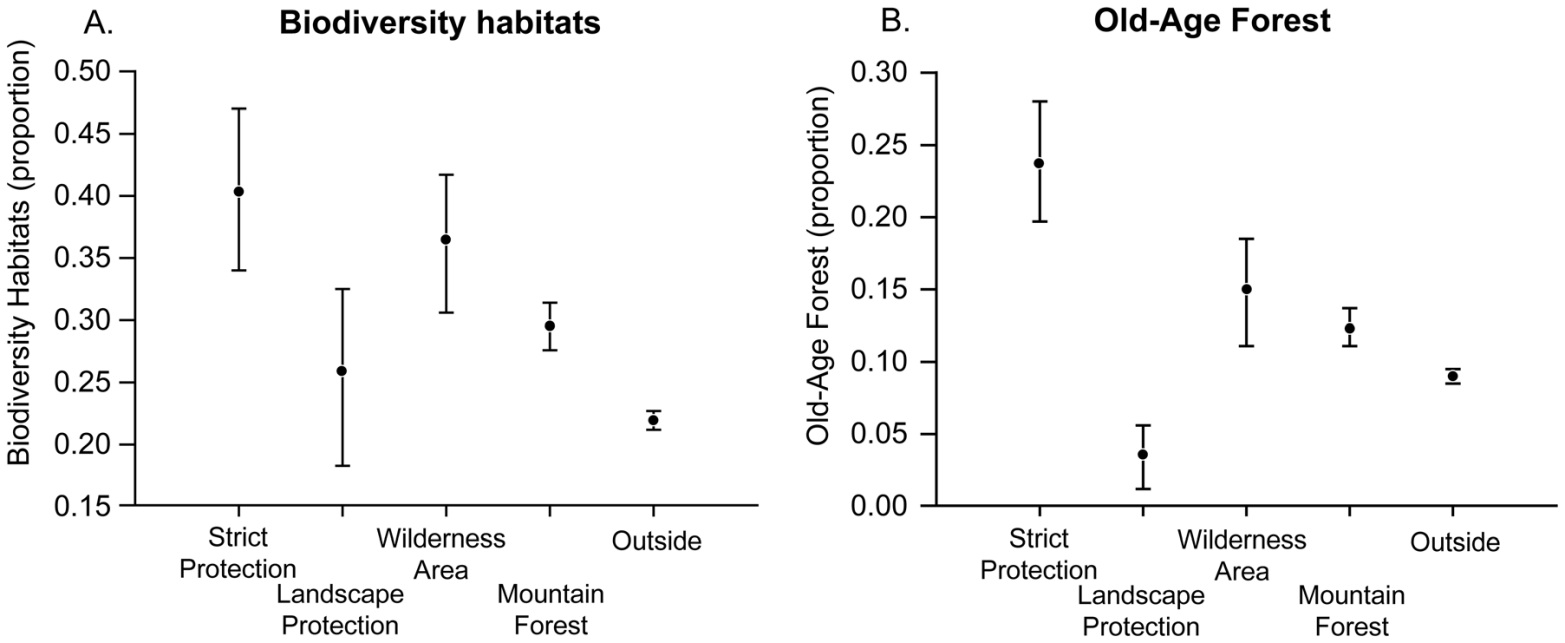
The proportion of A Biodiversity Habitats and B Old-Age Forest within forest targeted by each of the four large-scale policy instruments studied and outside (i.e. in forest not targeted by the large-scale instruments). Mean value (dots) and 95% Confidence Interval (lines) is given. For more detailed data, see Table 6.

The proportion of forest with Biodiversity Habitats was significantly higher in areas that coincided with Strict Protection, Wilderness and Mountain Forest, than in forest outside each of these areas (**Fig. 2**). When considering Biodiversity Habitats separately, we see that all these instruments except Landscape Protection targeted a higher proportion of logs (**Table 6**). In addition, forest inside Strict Protection areas also had a significantly higher proportion of snags than forest outside of Strict Protection areas (**Table 6**).

**Table 6:** Relative proportion of Biodiversity Habitats, separate habitats (Multi-BH: Multi-Biodiversity Habitats, Old-Age BHs: Old-Age Biodiversity Habitats) and Old-Age Forest within and outside forest targeted by each policy instruments.

	Strict Protection vs. Not					Landscape Protection vs. Not					Wilderness vs. Not					Mountain forest vs. Not				
	Mean	CI		Mean	CI	Mean	CI		Mean	CI	Mean	CI		Mean	CI	Mean	CI		Mean	CI
Biodiversity Habitat	**40,3%**	**[34,3**–**47,2]**	**vs.**	**21,8%**	**[21,2**–**22,5]**	25,9%	[18,3–32,5]	vs.	22,2%	[21,5–22,9]	**36,5%**	**[30,6**–**41,7]**	**vs.**	**21,9%**	**[21,3**–**22,6]**	**29,5%**	**[27,6**–**31,4]**	**vs.**	**20,8%**	**[20**–**21,7]**
Individual habitats:																				
Snags	**8,3%**	**[5,1**–**10,8]**	**vs.**	**2,5%**	**[2,3**–**2,7]**	1,7%	[0–2,9]	vs.	2,7%	[2,5–3]	5,7%	[4–7,4]	vs.	2,6%	[2,4–2,8]	3,1%	[2,4–3,7]	vs.	2,6%	[2,4–2,8]
Logs	**24,3%**	**[20,7**–**28,4]**	**vs.**	**13,1%**	**[12,6**–**13,5]**	19,3%	[11,7–25,4]	vs.	13,3%	[12,7–13,7]	**27,0%**	**[23,1**–**31,1]**	**vs.**	**12,9%**	**[12,4**–**13,5]**	**19,9%**	**[18**–**21,4]**	**vs.**	**12,0%**	**[11,6**–**12,6]**
Nutrient-rich bark	0,8%	[0–1,4]	vs.	0,2%	[0,2–0,3]	0%	[0–0]	vs.	0,2%	[0,2–0,3]	0,4%	[0–0,7]	vs.	0,2%	[0,2–0,3]	0,2%	[0–0,3]	vs.	0,2%	[0,2–0,3]
Pendant lichens	2,8%	[1,3–4,5]	vs.	2,8%	[2,5–3]	2,8%	[1,1–4,8]	vs.	2,8%	[2,5–3,1]	1,9%	[0,6–2,9]	vs.	2,8%	[2,6–3]	5,5%	[4,5–6,5]	vs.	2,2%	[2–2,5]
Late succ. broadl.	2,5%	[1,1–3,5]	vs.	1,5%	[1,3–1,7]	0,4%	[0–0,8]	vs.	1,5%	[1,4–1,7]	3,1%	[1,6–4,4]	vs.	1,5%	[1,3–1,7]	1,3%	[0,8–1,7]	vs.	1,6%	[1,4–1,8]
Old trees	5,7%	[3,6–8,1]	vs.	1,6%	[1,4–1,8]	0,9%	[0–1,7]	vs.	1,7%	[1,5–1,9]	3,1%	[1,5–4,5]	vs.	1,7%	[1,5–1,8]	2,8%	[2,1–3,3]	vs.	1,5%	[1,2-1,7]
Burned forest	0,4%	[0–0,7]	vs.	0,1%	[0,1–0,2]	0%	[0–0]	vs.	0,1%	[0,1–0,2]	0%	[0–0]	vs.	0,2%	[0,1–0,2]	0,2%	[0–0,3]	vs.	0,1%	[0–0,2]
Rich vegetation	3,8%	[1,9–5,9]	vs.	2,6%	[2,4–2,8]	3,2%	[1,2–5,4]	vs.	2,7%	[2,4–2,9]	1,1%	[0–1,8]	vs.	2,7%	[2,5–3]	0,6%	[0,3–0,9]	vs.	3,1%	[2,8–3,3]
Multi-BHs	**26,0%**	**[20,0**–**31,6]**	**vs.**	**9,6%**	**[9,0**–**10,1]**	6,8%	[2,3–10,7]	vs.	10%	[9,5–10,6]	**16,9%**	**[12,1**–**21,6]**	**vs.**	**9,8%**	**[9,3**–**10,4]**	**12,3%**	**[10,5**–**14,1]**	**vs.**	**9,6%**	**[9,0**–**10,2]**
Old-Age BHs	**16,4%**	**[12,2**–**20,1]**	**vs.**	**4,3%**	**[3,9**–**4,6]**	3,0%	[0–4,7]	vs.	4,6%	[4,2–5]	**9,7%**	**[6,8**–**12]**	**vs.**	**4,4%**	**[4**–**4,8]**	**7,2%**	**[6,1**–**8,3]**	**vs.**	**4,1%**	**[3,8**–**4,4]**
Old-Age Forest	**23,8%**	**[19,7**–**28]**	**vs.**	**9,0%**	**[8,5**–**9,5]**	3,5%	[1,2–5,6]	vs.	9,5%	[9–9,9]	**15,0%**	**[11,1**–**18,5]**	**vs.**	**9,3%**	**[8,9**–**9,8]**	**12,4%**	**[11,1**–**13,7]**	**vs.**	**8,8%**	**[8,3**–**9,3]**

Bold denotes significant p-value in Wilcoxon Rank Sum Test (p<0.05). 95% Confidence Interval is given in brackets.

Strict Protection forest also included almost four times the proportion of Old-Age Biodiversity Habitats as the forest not strictly protected (16% vs 4%), and more than double the proportion of Multi-Biodiversity Habitats (26% vs. 10%) and Old-Age Forest (24% vs. 8%). Also forest in Wilderness Areas and in Mountain Forest covered significantly higher proportions of Old-Age Biodiversity Habitats and Multi-Biodiversity Habitats than forest outside these areas (**Table 6**).

For Landscape Protection, the area was small (as indicated by the confidence intervals including zero) and none of the patterns proved significant (**Table 6**).

The proportion of Biodiversity Habitats was of similar magnitude in areas designated as Wilderness areas (37%) and in areas under Strict Protection (40%), with overlapping confidence intervals (**Fig. 2**). The same was true for Multi-Biodiversity Habitats. However, for Old-Age Forest and Old-Age Biodiversity Habitats, the proportion was higher in forest under Strict Protection than otherwise (**Table 6**).

### Overlap between instruments

The policy instruments targeting environmental protection in forest overlapped to a certain degree. **Table 7** shows that the greatest overlap between instruments involved Mountain Forest. Approximately 66% of both Landscape Protection areas and Wilderness Areas were also within Mountain Forest areas. Also, about 25% of both Strict Protection areas and Landscape Protection areas were also targeted by the Wilderness instrument (**Table 7**). In total, 3% of the productive forest is covered by two or more instruments, 16% is covered by one instrument and the remaining 80% is not covered by any of the instruments studied.

**Table 7:** Overlap (%) between different policy instruments.

	No overlap	Strict Protection	Landscape Protection	Wilderness	Mountain forest
Strict Protection	51%	-	0%	25%	40%
Landscape Protection	20%	0%	-	24%	68%
Wilderness	23%	22%	11%	-	64%
Mountain Forest	72%	6%	5%	11%	-

The sum of each row exceeds 100% in some cases as only overlaps between two instruments were considered.

A relevant question is the extent to which spatially overlapping policy instruments in forest correspond to higher conservation values. There was limited complementarity among instruments in terms of the proportion of Biodiversity Habitat and Old-Age forest representation. The only clear difference in terms of non-overlapping confidence intervals was between forest covered by a combination of Strict Protection, Mountain Forest and/or Wilderness Area, and forest covered by Mountain Forest alone (**Table 8**).

**Table 8:** Proportion of each instrument category that contain either Biodiversity Habitat or Old-Age Forest. 95% Confidence Interval given in brackets.

	Biodiversity Habitat	Old-Age Forest
Strict Protection	39% [32–47]	8% [0–14]
Strict Protection and Mountain Forest and/or Wilderness	43% [34–53]	2% [0–3]
Landscape Protection	27% [14–38]	12% [11–14]
Landscape and Mountain Forest and/or Wilderness	25% [16–35]	11% [6–15]
Mountain Forest	29% [26–31]	8% [8–9]
Wilderness	36% [23–46]	22% [17–28]
Mountain Forest and Wilderness	32% [25–41]	26% [19–33]

Forest covered only by Mountain Forest had a rather low proportion of Old-Age Forest, while the 11% (**Table 7**) of Mountain Forest that was also covered by the Wilderness Area instrument show a 3-fold increase in Old-Age forest (**Table 8**).

## Discussion

We studied how the four main large-scale instruments in the policy mix for forest conservation are implemented across the forest landscape in Norway, and the extent of overlap with forest of high conservation value. We found that the extensive area designated as Mountain Forest also covers the largest proportion of forest of high conservation value (**Fig. 1**), and that all policy instruments except Landscape Protection target a larger percentage of forest of high conservation value, compared to forest that is not under these conservation instruments (**Fig. 2**). The instrument with the highest restriction level (Strict Protection) had the highest overlap with Old-Age Forest (6,8%; **Table 5**) and the highest proportion of Old-Age Forest (23,8%; **Fig. 2**, **Table 6**). But, contrary to our hypothesis, the less strict instrument Wilderness Area targeted a similar proportion of Biodiversity Habitats as Strict Protection (4,9% vs. 4,9%; **Table 5**). Also, a comparably large portion of Biodiversity Habitats occurred in areas designated as Wilderness Areas and as Mountain Forest – for instance, 37% of the Wilderness Areas consisted of Biodiversity Habitats (**Fig. 2**, **Table 6**).

### The significance of Old-Age Forest

As clear-cutting was introduced to Norway only 60–70 years ago, the Old-Age Forest in this study has never been clear-cut, but has most likely experienced selective cuttings of various intensities. Still, a continuous forest cover, regeneration through local seed sources, and dispersed biological legacies like old trees are important qualities in this forest. With time, amounts of dead wood will increase [28] and enhance its conservation value. An importance of Old-Age Forest for forest conservation is further strengthened by our finding of a considerably overlap between Biodiversity Habitats and Old-Age Forest. The high frequency of Biodiversity Habitats in Old-Age Forest and the separate qualities described above emphasizes the need for policy instruments to safeguard the conservation qualities in Old-Age Forest. At present, more than 70% of this forest is not covered by any of the studied instruments.

Not all ecological qualities in forests coincide with old age. The early, open successional stages after natural disturbances like forest fire or windstorms also provide habitats of high importance for forest biodiversity [29], [30]. These qualities are at risk due to interference with the natural forest disturbance and succession dynamics, including containment of forest fires, salvage logging in the few wind-felled or burnt areas that occur, and the practice of planting coniferous species. The habitats “Late successional broadleaf forest” and “Burned forest” describe these qualities, but we found no significant trends here. Even in our extensive dataset these habitats are rare, which makes possible patterns hard to detect.

### Amount of protection

We have considered the main instruments targeting large-scale conservation in forest, and show that more than 70% of both Biodiversity Habitats and Old-Age Forest is presently not targeted by any of these. So what amount of forest of high conservation value would be necessary to protect, for effective conservation outcomes relative to conservation policy objectives? This of course depends on a number of issues, including the complementarity of the habitats protected, the amount and effectiveness of supplementary small-scale instruments, the restriction level of the instruments and the related long-term conservation effectiveness. This will be discussed in brief below. Some habitats are rare and might need a higher protection level than others [31]. Also, a habitat type will have different species composition depending on environmental gradients and site-specific factors. This needs to be addressed in an evaluation of conservation level.

Some of the forest area outside the area targeted by large-scale instruments will be protected or partially protected by guidelines in the forest certification standards addressing small-scale conservation measures, also contributing to the conservation of biodiversity [16]. This includes setting aside Woodland Key Habitats, retaining buffer zones in riparian forest and a number of trees in the harvesting units, and avoiding clear-cutting in swamp forest [12], [32]. Although research is accumulating on the effects of such harvesting prescriptions, little is known about the accumulated effects of these measures after several forest rotations.

### Effectiveness of policy instruments

We have studied the amount of overlap between the policy tools and forest of high conservation value, but area coverage is not synonymous with conservation effectiveness. Another important aspect is the potential of the instruments to ensure long-term persistence of the conservation features. This question has two dimensions. One is the long-term, accumulated effect of the harvesting activities permitted. For instance, while logging is banned in areas under Strict Protection, it is allowed with some restrictions under the other three policy instruments. With time and accumulating effects of forestry, this may potentially reduce the amount and qualities of Old-Age Forest and Biodiversity Habitats in these areas.

The other dimension is the permanence of the policy tool. The Wilderness Area instrument is a good example. This instrument originated as a monitoring tool to quantify the decline of larger areas without infrastructure, and has gradually turned into a policy instrument [24], [25]. In the case of forest protection, this development can be seen in a regulation under the Forestry Act, stating that subsidies for permanent road building will not to be granted in cases where this reduces Wilderness Areas. Hence, even though no restrictions apply to logging in Wilderness Areas, without subsidies it is normally not profitable and therefore forest exploitation rarely takes place. The instrument of Wilderness Area has been much discussed, both regarding the processes leading to the present use and not the least questioning its importance for biodiversity conservation [33]. This study is the first to document the large proportion of forest of high conservation value in Wilderness Areas, although it has been suggested in earlier reports [34]. Also, a recent study from Sweden shows that the length of forest roads in the landscape is negatively correlated with abundance and occurrence of red-listed wood-inhabiting fungi [35]. The present Norwegian government has decided to discontinue the use of the Wilderness criterion as a policy tool for all sectors, including forestry. This is expected to make wilderness areas eligible for forest road construction subsidies, illustrating the volatility of policy instruments in relation to a policy objective of long term forest conservation.

### Overlap between instruments

An evaluation of overlap between the policy instruments can address their simple spatial coincidence, overlap in specific forestry restrictions, and finally, whether overlap results in any effects on conservation effectiveness.

As for the spatial extent, the different instruments are not fully complementary. Approximately 3% of the forest area is targeted by more than one policy instrument. In some cases the overlap is substantial: The overlap between Mountain Forest and all the other instruments is more than 40%, and 25% of Wilderness Areas overlap with the regulatory instruments. There are logical reasons that can explain much of this overlap. For instance, Strict Protected areas are often situated far from infrastructure and buildings, and thus they might also qualify as Wilderness Areas. Similarly, National Parks, mostly situated in the alpine areas of Norway, will overlap with the high-altitude forest that Mountain Forest is intended to cover.

Another issue is whether the instruments are complementary, conflicting or redundant in terms of restrictions to forestry. Strict Protection permits no forestry activities, while both Landscape Protection and Mountain Forest permit forestry with certain limitations. These limitations may differ, as the protection aims are different. In our study we find no difference in the proportion of Biodiversity Habitat or Old-Age Forest in combinations of Landscape Protection and Mountain Forest compared to alone (**Table 8**).

### Ambitions and opportunities to improve the current policy mix

The international Aichi Biodiversity Targets state that 17% of the areas of particular importance for biodiversity and ecosystem services are to be conserved, through “ecologically representative and well-connected systems of protected areas and other effective area-based conservation measures” (Convention on Biological Diversity). Previous evaluations in Norway have suggested a minimum of 5% strict protection in forest, and a future goal of 10% [36].

Our results show that the mix of large-scale conservation instruments in Norway covers 23% of the productive forest area. It also reveals that even though the current policy mix has an above average representation of high conservation value forests, the targeting effectiveness when it comes to forest with high conservation value is limited. This corresponds to findings by Schröter et al. (this issue) for Telemark County. Hence, our results are in line with Framstad et al. [36] indicating both the need to improve forest conservation and a potential to cover this need by better targeting forest of high conservation value.

The type of policy instruments necessary to improve the current policy mix is not clear, but our analyses indicate some potential directions. Policy instruments focusing specifically on protecting biologically old forest are currently lacking, although this forest contains a high proportion of areas with forest of high conservation value. As more than 70% of the area with habitats important for red-listed forest species is not targeted by any of the studied policy instruments, one possibility is to increase efficiency in the allocation of conservation resources when regulating forests for Strict Protection. The present Norwegian Voluntary Forest Conservation Scheme could for example be used to target areas with particularly high conservation value for protection [27]. In this context, Meir et al. [37] found that paying a premium to land-owners could enhance cost-effectiveness by increasing the offer of potential sites to protect.

Also, there seems to be opportunities to adapt and target additional regulation within Wilderness Area and Mountain Forest to protect forest of high conservation value where these forms of protection may be insufficient to ensure long-term persistence due to policy volatility.

In conclusion, we found that the proportion of areas of high conservation value was higher within areas targeted by policy instruments, than outside such areas, except for areas under Landscape Protection. We further found that areas targeted by Strict Protection contained the highest proportion of Old-Age Forest, while for Biodiversity Habitats, areas targeted by Wilderness Area showed similar proportions. Finally, we found a substantial amount of spatial overlap between the policy tools, but no conservation effect of overlapping instruments in terms of contributing to higher proportions of areas of high conservation value.
